# Cigarette smoke increases cardiomyocyte ceramide accumulation and inhibits mitochondrial respiration

**DOI:** 10.1186/1471-2261-14-165

**Published:** 2014-11-22

**Authors:** Trevor S Tippetts, Duane R Winden, Adam C Swensen, Michael B Nelson, Mikayla O Thatcher, Rex R Saito, Tyler B Condie, Kurtis J Simmons, Allan M Judd, Paul R Reynolds, Benjamin T Bikman

**Affiliations:** Department of Physiology and Developmental Biology and Chemistry, Brigham Young University, Provo, UT 84602 USA; Department of Chemistry and Biochemistry, Brigham Young University, Provo, UT 84602 USA

## Abstract

**Background:**

Cigarette smoking is a common and lethal worldwide habit, with considerable mortality stemming from its deleterious effects on heart function. While current theories posit altered blood lipids and fibrinogen metabolism as likely mediators, none have explored the role of the sphingolipid ceramide in exacerbating heart function with smoke exposure. Ceramide production is a consequence of cigarette smoke in the lung, and considering ceramide’s harmful effects on mitochondrial function, we sought to elucidate the role of ceramide in mediating smoke-induced altered heart mitochondrial respiration.

**Methods:**

Lung cells (A549) were exposed to cigarette smoke extract (CSE) and heart cells (H9C2) were exposed to the lung-cell conditioned medium. Adult male mice were exposed sidestream cigarette smoke for 8 wk with dietary intervention and ceramide inhibition. Ceramides and heart cell or myocardial mitochondrial respiration were determined.

**Results:**

Lung cell cultures revealed a robust response to cigarette smoke extract in both production and secretion of ceramides. Heart cells incubated with lung-cell conditioned medium revealed a pronounced inhibition of myocardial mitochondrial respiration, though this effect was mitigated with ceramide inhibition via myriocin. In vivo, heart ceramides increased roughly 600% in adult mice with long-term sidestream cigarette smoke exposure. This resulted in a significant ceramide-dependent reduction in left myocardial mitochondrial respiration, as heart mitochondria from the mice exposed to both smoke and myriocin injections respired normally.

**Conclusions:**

These results suggest ceramide to be an important mediator of altered myocardial mitochondrial function with cigarette smoke exposure. Thus, anti-ceramide therapies might be considered in the future to protect heart mitochondrial function with smoke exposure.

## Background

Cigarette smoke exposure is the leading cause of preventable deaths worldwide [1] and is among the top ten contributors to the worldwide health burden [2]. Despite concerted social efforts to reduce smoking prevalence, current trends suggest the number of smokers will increase worldwide [3, 4]. Moreover, cigarette smoke is a common inhaled toxin—almost half of the U.S. population is regularly exposed to cigarette smoke [5, 6] and approximately 20% of young children live with someone who smokes in the home [7]. Much of smoking’s health burden stems from the increased risk of chronic diseases like cancer, emphysema, and cardiovascular disease [8, 9], including cardiomyopathy—a deterioration of heart muscle.

Cardiomyocytes are highly oxidative cells with a tremendous reliance on mitochondrial capacity [10], and altered mitochondrial function can lead to heart failure [11–13], a common consequence of cardiomyopathy. Considering the importance of healthy mitochondrial function in cardiomyocyte homeostasis, a valuable area of study is to elucidate the factors that mediate altered heart mitochondrial physiology and its effects with cigarette smoke exposure. Previous studies have observed that cigarette smoke exposure inhibits mitochondrial respiratory function in blood cells [14] and myocardium [15], but a mediating mechanism has yet to be identified.

Cigarette smoke has long been known to robustly activate inflammatory pathways in the lung [16], which increases ceramide biosynthesis [17, 18]. Importantly, ceramides are known to disrupt mitochondrial structure and function [19, 20], possibly increasing risk of cardiomyopathy [21]. Thus, the purpose of these studies was to determine whether the sphingolipid ceramide mediates cardiomyocyte mitochondrial disruption with cigarette smoke exposure. Considering the lung’s apposition with the environment, the lung is a logical site of external pathogen-induced stress, a product of which is ceramide biosynthesis [17]. Moreover, given the heart’s location relative to pulmonary blood flow, the heart is a reasonable site of lung-derived ceramide uptake.

## Methods

### Cell culture

Cigarette smoke extract (CSE) was generated as previously described with slight modifications [22]. Briefly, one 2RF4 research cigarette (University of Kentucky, Lexington, KY) was continuously smoked by connecting the filtered end of the cigarette to a vacuum pump, pulling the particles into 5 ml of DMEM/F12 and the resulting medium was defined as 100% CSE. The total particulate matter content of 2RF4 cigarettes is 11.7 mg/cigarette, tar is 9.7 mg/cigarette, and nicotine is 0.85 mg/cigarette. Dilutions were made using DMEM/F12 + 10% FBS. Human type II–like pulmonary adenocarcinoma cells (A-549; passage 10-15) were maintained in DMEM/F12 supplemented with 10% FBS (Invitrogen) and antibiotics. Cells were split into 6-well dishes and grown to 80% confluence. H9C2 cardiomyocytes were maintained in DMEM +10% FBS. For differentiation into myotubes, cells were grown to confluency and the medium was replaced with DMEM +10% horse serum (Invitrogen, Grand Island, NY). Myotubes were used for experiments on day 3 of differentiation. A-549 cultures were exposed to media supplemented with 10% CSE or media alone for 4 h, after which the medium was transferred to differentiated H9C2 cardiomyocytes (termed “conditioned medium”) for 12 h. Where indicated, cells were treated with myriocin (10 μM, Sigma, M1177), a known and widely used inhibitor of ceramide biosynthesis. Muscle cells were harvested for RNA, protein, and lipid isolation following treatments. Cell survival during treatments was confirmed by trypan blue exclusion.

### Animals

Male C57Bl6 mice were housed in a conventional animal house and maintained on a 12-hour light–dark cycle. Two animal studies were conducted. In the first study, animals received standard diet chow (Harlan Teklad 8604) and water *ad libitum*. At 12 wk of age, animals were randomly divided into room air and cigarette smoke (CS)-exposed groups. Mice were placed in soft restraints and connected to the exposure tower of a nose-only exposure system (InExpose System, Scireq, Canada). Animals were nasally exposed to sidestream CS generated by research cigarettes where a computer-controlled puff was generated every minute, leading to 10 min of CS exposure followed by 10 min of fresh air, repeated once. The CS-exposed group inhaled CS from two cigarettes per day for one week, at which point the dosage was increased to two cigarettes twice daily. Control animals were similarly handled and restrained in fresh air for the same duration. For the second study, 12-wk-old mice were separated into one of eight treatment groups for eight weeks. Each of the following groups were duplicated to have one group receive vehicle or myriocin injections (0.3 mg/kg) every other day for a total of eight groups: 1) control – standard diet chow, no smoke; 2) cigarette smoke exposure (two cigarettes, twice daily) with standard diet chow; 3) high-fat, high-sugar diet (Harlan Teklad 45F30S); 4) high-fat, high-sugar diet with cigarette smoke exposure. Tissues were harvested at the conclusion of the study period. Studies were conducted in accordance with the principles and procedures outlined in the National Institutes of Health Guide for the Care and Use of Laboratory Animals and were approved by the Institutional Animal Care and Use Committee (IACUC) at Brigham Young University.

### Lipid analysis

For isolation of lipids, cell pellets or tissue were suspended in 900 μl ice-cold chloroform/methanol (1:2) and incubated for 15 minutes on ice, then briefly vortexed. Separation of aqueous and organic phases required addition of 400 μl of ice-cold water and 300 μl of ice-cold chloroform. The organic phase was collected into a fresh vial, and lipids were dried in a vacuum centrifuge (Eppendorf Concentrator Plus). Lipids were characterized and quantified using a shotgun lipidomics technique on a Thermo Scientific LTQ Orbitrap XL mass spectrometer. Evaporated lipid samples were re-suspended in a 2:1 chloroform: methanol Folch solution (200 μL). The re-suspended lipids were then combined with a modified 2:1:1.25 chloroform: methanol: isopropanol Bligh and Dyer solution (800 μL) with 15 mM ammonium acetate acting as an ionizing adduct. A 1.74 μM phosphatidylethanolamine, 1 μM C-17 ceramide, 1 μM tripalmitin internal standard cocktail (1 μL) was spiked into each sample for mass calibration and characterization data alignment. Samples were analyzed using a 2.5-minute mass-window scanning method in positive-ion mode at a resolution of 100,000 (FWHM at 400 m/z) for all primary MS^1^ scans. MS^2^ fragmentation data was also collected (PQD at relative intensity of 35) and manually verified for each mass window to give additional confidence to the correct identification of abundant lipid species. Three technical replicate mass spectrometer runs were performed on each sample. Samples were injected at 8 μL/min using a direct-inject electrospray ionization (ESI) soft-ionization spray head from a Hamilton GASTIGHT glass syringe. A nitrogen sheath gas spray flow rate of 8 (arb. Units) was used for all runs. The spray voltage and capillary temperature were maintained at 5.0 KV and 275°C respectively. Each technical replicate was run in random order to reduce systematic bias. Data were analyzed using in-house developed peak summarization, recalibration, and lipid identification software using lipid database information from the LIPID Metabolites and Pathways Strategy (Lipid MAPS) Lipidomics Gateway database [23]. To ensure high-confidence identifications, an intensity threshold estimated to be 5% above instrumental static signal was implemented. Lipid identities were only assigned when significantly observable peaks were identified in at least two of the three technical replicate runs. Non-zero lipid quantities were averaged from the replicate runs. The lipid species identified across different ionization states or with adducts were totaled together. Quantification was completed by normalizing total ion counts to the relative abundance of the internal standard that was spiked into each sample.

### Cell and myocardium permeabilization

For cells, H9C2 cardiomyocytes were detached in culture dishes with 0.05% trypsin-EDTA (Sigma) and growth medium was added to the culture. Contents were transferred to a tube and centrifuged for 10 min at 1000 × *g* at RT. After removal of supernatant, cells were lifted in MiR05 [0.5 mM EGTA, 3 mM MgCl_2_, 60 mM K-lactobionate, 20 mM taurine, 10 mM KH_2_PO_4_, 20 mM HEPES, 110 mM sucrose, and g/l BSA (Sigma; A3803) adjusted to pH 7.1] plus 1 mg/ml digitonin and gently rocked at RT for 5 min before centrifugation at 1000 × *g* for 5 min. After discarding supernatant, cells were then suspended in 2.2 ml warm MiR05 and transferred to chambers in the O2K (Oroboros Instruments, Innsbruck, Austria). Following respiration protocol (outlined below), cells were removed from the chambers and used for further analysis, including protein quantification. For myocardial mitochondrial respiration, left ventricle was quickly removed from euthanized mice and immediately placed in ice-cold buffer X (60 mM K-MES, 35 mM KCl, 7.23 mM K_2_EGTA, 2.77 mM CaK_2_EGTA, 20 mM imidazole, 20 mM tuarine, 5.7 mM ATP, 15 mM PCr, 6.56 mM MgCl_2_-6H_2_O, pH 7.1) and trimmed of connective tissue. Small fiber bundles were prepared and gently separated along their longitudinal axis under a surgical scope (Olympus, ST) to 1-2 mg. Bundles were then transferred to a tube with chilled buffer X and 50 μg/ml saponin and rocked at 4°C for 30 min, then washed in buffer Z (105 mM K-MES, 30 mM KCl, 10 mM KH_2_PO_4_, 5 mM MgCl_2_-6H_2_O, 0.5 mg/ml BSA, pH 7.1) at 4°C for at least 15 min. Samples were then blotted dry and weighed.

### Mitochondrial isolation

To isolate mitochondria from left ventricle myocardium, the whole heart was placed in isolation buffer (300 mM sucrose, 10 mM Na-HEPES, 0.2 mM EDTA, pH 7.2) and the left ventricle isolated. After finely mincing the sample, trypsin (Sigma T9935) was added for two minutes before adding trypsin inhibitor (Sigma T9003). The sample was then transferred to a conical tube and allowed to settle. After removal of supernatant, samples were resuspended in isolation buffer with BSA (Sigma A3803) and homogenized with Teflon pestle before centrifugation (600 × *g* for 10 min at 4°C). Supernatant was then transferred to a new tube and centrifuged (8000 × *g* for 15 min at 4°C), then washed by gently adding isolation buffer and rotating the tube. A portion of this suspension is used for protein quantification. The pellet was then resuspended with isolation buffer + BSA.

### Mitochondrial respiration protocol

High-resolution O_2_ consumption was determined at 37°C in permeabilized cells and fiber bundles using the Oroboros O2K Oxygraph with MiR05 respiration buffer. Before addition of sample into respiration chambers, a baseline respiration rate was determined. After addition of sample, the chambers were hyperoxygenated to ~350 nmol/ml. Following this, respiration was determined by all or parts of the following substrate-uncoupler-inhibitor-titration (SUIT) protocol [24]: electron flow through complex I was supported by glutamate + malate (10 and 2 mM, respectively) to determine basal oxygen consumption (GM_*B*_). Following stabilization, ADP (2.5 mM) was added to determine oxidative phosphorylation capacity (GM_*P*_). Succinate was added (GMS_*P*_) for complex I + II electron flow into the Q-junction. To determine full electron transport system (F) capacity over oxidative phosphorylation, the chemical uncoupler carbonyl cyanide 4-(trifluoromethoxy) phenylhydrazone (FCCP) was added (0.05 μM, followed by 0.025 μM steps until maximal O_2_ flux was reached). Complex II-supported ETS was then measured by inhibiting complex I with rotenone (Rot; 0.5 μM). Mitochondrial membrane integrity was tested in all experiments by adding cytochrome *c* (10 μM; GMc_*P*_). Lastly, residual oxygen consumption was measured by adding antimycin A (2.5 μM) to block complex III action, effectively stopping any electron flow, which provides a baseline rate of respiration. Where indicated, C2-ceramide (Sigma A7191; 20 μM) was added to respiration chambers.

### Statistics

Data are presented as the mean ± SEM. Data were compared by ANOVA with Tukey’s post-hoc analysis (Graphpad Prism; La Jolla, CA). Significance was set at *p* <0.05.

## Results

### Ceramide inhibits cardiomyocyte mitochondrial respiration

**Figure 1:**
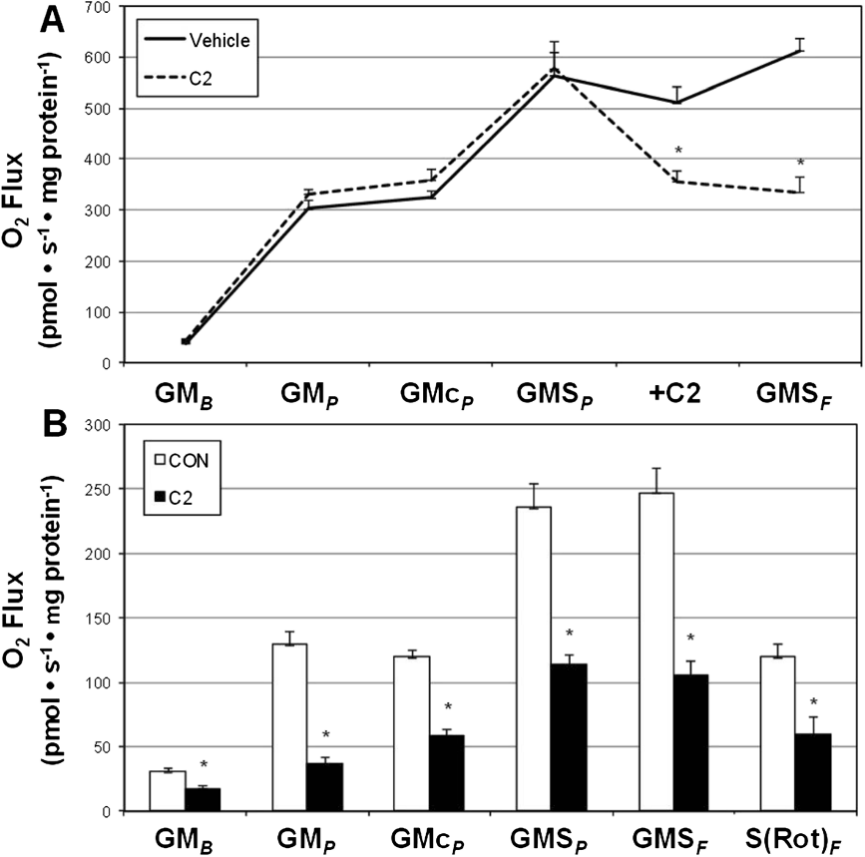
**Ceramide inhibits left ventricle mitochondrial respiration.** ***A***: Respiration rates of mitochondria isolated from left ventricle myocardium with addition of C2-ceramide during respiration protocol (20 μM; n = 4). ***B***: Mitochondrial respiration from permeabilized left ventricle myocardium (30 min) with continuous incubation with C2-ceramide (20 μM; n = 8). See Methods for more details on respiration protocol. *P <0.05.

To determine the effects of ceramide on cardiac mitochondrial respiration, we utilized two models. First, mitochondria were isolated from left ventricle myocardium. During the course of the respiration protocol in the isolated mitochondria, C2-ceramide was added to one oxygraph chamber. The addition of ceramide (+C2) elicited a rapid and significant reduction in mitochondrial respiration that was sustained through maximal respiration with FCCP (Figure 1A; see Methods for protocol details). A second model was the use of permeabilized intact left ventricle myocardium. One sample was incubated with C2-ceramide throughout the length of the protocol, which elicited a sustained reduction in mitochondrial respiration throughout the entire protocol (Figure 1B).

### Cigarette smoke extract increases lung cell ceramide production and secretion

**Figure 2:**
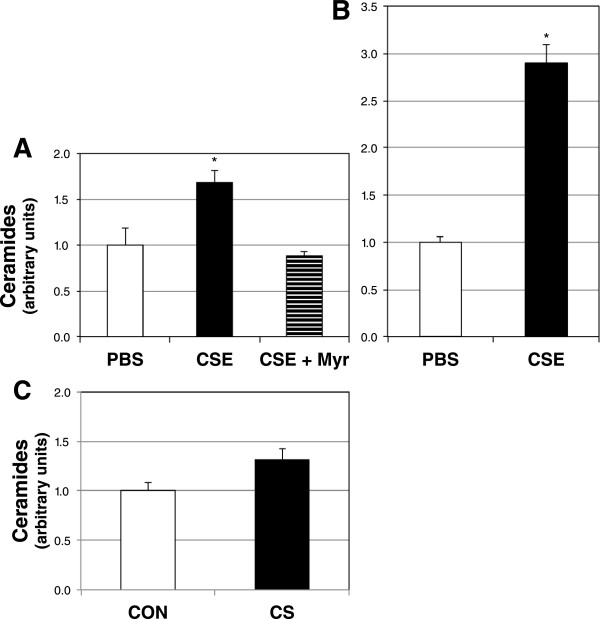
**Lung cells make and secrete ceramide in response to cigarette smoke extract.** ***A***: Ceramide levels in A549 cells treated with PBS- or 10% cigarette smoke extract-containing medium without (CSE) or CSE with myriocin (CSE + Myr), a ceramide inhibitor, for 12 h (n = 6). ***B***: Ceramides in culture medium of A549 cells following a 12-h treatment with PBS or CSE (n = 6). ***C***. Ceramides were determined from whole blood of adult mice following 5 d of room air (CON) or sidestream cigarette smoke (CS) (*P* =0.069; n = 5). *P <0.05 for CSE vs. PBS.

The deleterious effect of cigarette smoking on heart function is undeniable, but the mechanism communicating the pulmonary insult to the heart is not clear. As proof of concept that the lung is capable of producing and secreting ceramides with smoke exposure, we treated A549 alveolar type 2 cells with cigarette smoke extract (CSE). Relative to PBS treatment, ceramides increased over 60% in CSE-treated lung cells, though this effect was mitigated with myriocin co-treatment (Figure 2A). Further, CSE treatment elicited an almost three-fold increase in ceramide secretion into medium compared with PBS treatment (Figure 2B). We also observed an upward trend (*P* = 0.069) in circulating ceramides from whole blood following 5 d of sidestream smoke exposure in adult mice (Figure 2C).

### Ceramide is necessary for smoke extract-induced altered cardiomyocyte mitochondrial disruption

**Figure 3:**
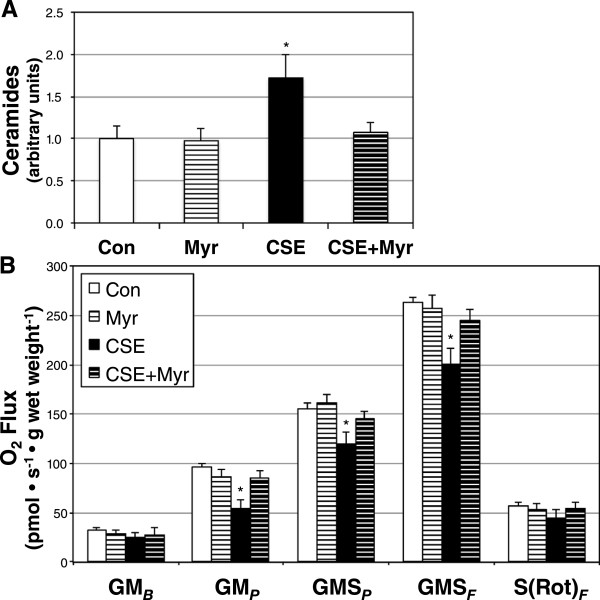
**Ceramide is necessary for decreased mitochondrial respiration in myocardial cells following treatment with conditioned medium from CSE-treated lung cells.** ***A***: Ceramide levels in H9C2 cardiomyocytes treated with conditioned medium from A549 alveolar type 2 cells following incubation with normal growth medium (Con), Con with myriocin (Myr), cigarette smoke extract (CSE), and CSE with Myr (CSE + Myr) (n = 4). ***B***: Mitochondrial respiration from H9C2 cardiomyocytes following treatment in identical conditions (n = 5). See Methods for more details on respiration protocol. *P <0.05 for CSE vs. Con.

To determine the effects of lung cell-secreted ceramides on heart cell function, we utilized a conditioned medium *in vitro* model. Briefly, following incubation with PBS- or CSE-containing medium, with or without myriocin co-treatment, the conditioned medium from lung cells was transferred to H9C2 cardiomyotubes for 12 h. Following the incubation with conditioned medium, we determined cardiomyotube ceramide accrual and mitochondrial respiration. Ceramides were significantly increased in cardiomyotubes treated with conditioned medium from CSE-treated lung cells (Figure 3A). However, when lung cells received treatment with myriocin in addition to CSE, the conditioned medium had no effect on heart cell ceramides (Figure 3A). Mitochondrial respiration in the heart cells followed a similar trend as ceramides. Namely, respiration in heart cells receiving CSE conditioned medium was negatively affected, but not when myriocin was included in the lung cell culture medium (Figure 3B).

### Ceramide inhibition prevents reduced myocardial mitochondrial respiration with cigarette smoke

**Figure 4:**
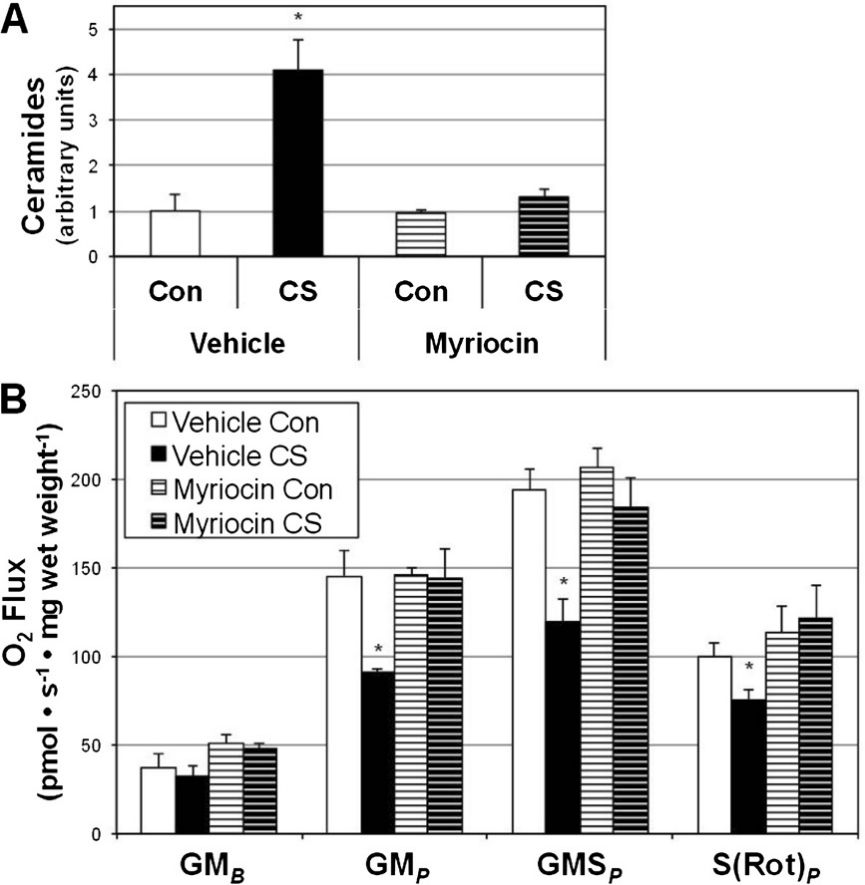
**Myriocin prevents left ventricle ceramide accrual and mitochondrial dysfunction with cigarette smoke.** Mice were exposed to room air (Con) or cigarette smoke (CS) for 1 wk while receiving PBS (vehicle) or myriocin injections every other day. ***A***: Ceramides were measured from left ventricle following treatment period (n = 6). ***B***: Mitochondrial respiration was reduced with CS treatment in vehicle-injected animals (n = 6). *P <0.05 for CS vs all other treatments.

To more accurately determine the role of ceramides in mediating smoke-induced decayed heart mitochondrial respiration, we exposed animals to CS for 1 wk while receiving injections of PBS (vehicle) or myriocin every other day. Left ventricle ceramides increased four fold with CS compared with room air-exposed mice (Figure 4A) with vehicle injections, though myriocin prevented this effect. Moreover, respiration was protected from CS in myriocin-injected animals (Figure 4B).

**Figure 5:**
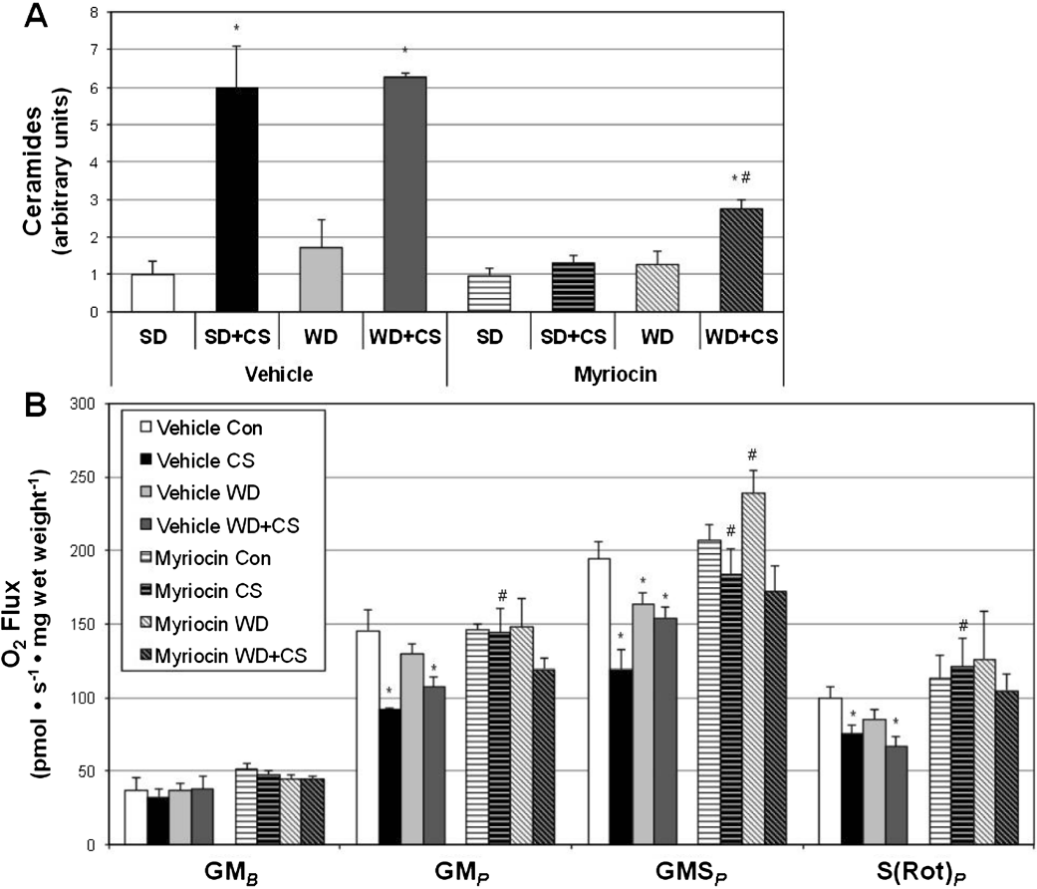
**Western diet exerts a minimal effect on heart ceramides in smoke-exposed mice.** Mice were exposed to room air (Con) or cigarette smoke (CS) for 8 wk while receiving PBS (vehicle) or myriocin injections every other day. Mice also received either a standard diet (SD) or Western diet (WD). ***A***: Ceramides were measured from left ventricle following treatment period (n = 4). ***B***: Mitochondrial respiration was determined from permeabilized left ventricle myocardium (n = 8). *P <0.05 for treatment vs. Vehicle SD. #*P* <0.05 for WD + CS myriocin vs. WD + CS vehicle.

Given the evidence suggesting the role of diet in increasing heart complications [25] and ceramide accrual [26], we provided a high-fat, high-sugar (Western diet; WD) to animals in conjunction with smoke (or room air) exposure over the course of an 8-wk study. Similar to before, animals received vehicle (PBS) or myriocin injections every other day. In addition to observing a roughly 6-fold increase in heart ceramides with smoke exposure, we found an equally great increase in heart ceramides in animals receiving WD diet and smoke (Figure 5A). However, ceramide inhibition was only partially successful in the WD + CS group. In general, ceramide accrual was associated with reduced myocardial mitochondrial respiration (Figure 5B).

## Discussion

Previous research demonstrated that smoke exposure contributes to cardiomyopathy [27, 28], which can be a consequence of altered mitochondrial function [21], and we have recently shown that sidestream smoke alters mitochondrial function in skeletal muscle [29]. Considering current worldwide smoking trends [3, 4], cardiomyopathy and other cardiovascular burdens mediated by cigarette smoke are likely to increase. To date, the main instigators thought to mediate the heart-specific effects of smoking are altered blood lipids and changes in fibrinogen metabolism, [30, 31], though the actual impact of these mechanisms is unknown [30]. While ceramides are known to mediate cellular disruption in the lung with smoking [32], its impact on cardiomyocyte function with smoking has not been adequately explored. Thus, the purpose of this project was to determine the role of tobacco smoke-induced ceramides in disrupting cardiomyocyte mitochondrial function. Our major discoveries were that lung cells secrete ceramide with sidestream smoke exposure and that ceramide accumulates in heart tissue and alters mitochondrial function.

To our knowledge, the first study to explore the effects of ceramide on mitochondrial respiration was published by the Hoppel laboratory, where they observed a rapid and robust inhibition of respiration in isolated heart mitochondria upon ceramide treatment [20], and subsequent work corroborates these findings [33]. We confirm those observations by Gudz et al. [20] in isolated heart mitochondria and report similar findings in permeabilized left ventricle. Additionally, we have previously shown that ceramides substantially inhibit complex II action and that the general adverse effect of ceramides on mitochondrial respiration is dependent on ceramide-induced mitochondrial fission [24]. Similarly, our findings of ceramide accrual in the lung with smoking corroborate those from other laboratories [34–36], but while previous work focused on ceramide generation via sphingomyelinase, our effective use of myriocin suggests the importance of *de novo* ceramide synthesis in sidestream smoke-induced ceramide accumulation. However, while myriocin injections were sufficient to completely block ceramide accrual with smoking or diet separately, it was insufficient to prevent an increase in ceramides with combined smoking and diet. This may be a result of insufficient myriocin action in the midst of an overpowering stimulus (diet and smoke combined), or that sphingomyelinase may be particularly relevant in our combined treatment.

Importantly, we not only find increased ceramide production in lung cells with smoke exposure, but also ceramide release, providing proof of concept that the lung may be at least a source of systemic ceramide accrual with smoke exposure. This is supported by our finding of an upward trend in circulating ceramides with smoke exposure. Nonetheless, ongoing experiments are testing the hypothesis that secreted ceramide by smoke-exposed pulmonary tissues travel and accumulate in cardiac muscle. Related to this, our conclusions that ceramide is the relevant component within the cultured medium upon transfer of medium from lung cells to cardiomyocytes is based on our use of myriocin. However, due to the harmful cocktail of molecules within the CSE, it is possible that a non-ceramide variable exists.

The level of ceramide accumulation we observe in the heart with smoking is substantial. We have previously quantified ceramide in various tissues (i.e., skeletal muscle, liver, brain) with dietary intervention [17, 24, 37] and rarely observed greater than a roughly twofold increase in ceramides; similar changes have been observed in the heart [26]. However, cigarette smoking appears to be a more robust inducer of systemic ceramide accumulation compared with diet. We found a roughly four-fold increase in heart ceramides after only 1 wk of smoke exposure (Figure 4) that increased to a six-fold change with an 8-wk exposure (Figure 4A). Interestingly, supplementing the smoking regimen with a dietary component (Western diet, Harlan Teklad 45F30S) had no additive effect on heart ceramides (Figure 5A).

## Conclusions

In conjunction with our recent findings of altered skeletal muscle function with cigarette smoke exposure [29], the results of these studies implicate ceramide as an important mediator of myriad systemic metabolic effects. In particular, we find evidence that ceramides are a mediator of sidestream smoke-induced altered heart mitochondrial function. While interventions to promote smoking cessation should continue, the increase in worldwide smoking and cardiovascular complications highlights the need for immediate therapies. Our findings suggest that ceramide inhibition may be a novel and potentially valuable therapeutic modality to protect heart function for those who are unwilling or unable to vacate smoke environments.
